# Automatic treatment planning for cervical cancer radiation therapy using direct three‐dimensional patient anatomy match

**DOI:** 10.1002/acm2.13649

**Published:** 2022-05-30

**Authors:** Duoer Zhang, Zengtai Yuan, Panpan Hu, Yidong Yang

**Affiliations:** ^1^ Department of Engineering and Applied Physics University of Science and Technology of China Hefei Anhui China; ^2^ Department of Radiation Oncology, the First Affiliated Hospital of USTC, Division of Life Sciences and Medicine University of Science and Technology of China Hefei Anhui China

**Keywords:** 3D contour registration, anatomical similarity, automatic treatment planning, cervical cancer radiation therapy

## Abstract

**Purpose:**

Current knowledge‐based planning methods for radiation therapy mainly use low‐dimensional features extracted from contoured structures to identify geometrically similar patients. Here, we propose a knowledge‐based treatment planning method where the anatomical similarity is quantified by the rigid registration of the three‐dimensional (3D) planning target volume (PTV) and organs at risks (OARs) between an incoming patient and database patients.

**Methods:**

A database that contains PTV and OARs contours from 81 cervical cancer radiation therapy patients was established. To identify the anatomically similar patients, the PTV of the new patient was registered to each PTV in the database and the Dice similarity coefficients were calculated for the PTV, rectum, and bladder between the new patient and database patients. Then the top 20 patients in the PTV match and top 3 patients in the subsequent bladder or rectum match were selected. The best dose–volume histogram parameters from the top three patients were applied as the dose constraints to the automatic plan optimization. A fast Fourier transform algorithm was developed to accelerate the 3D PTV registration process run through the database. The entire treatment planning process was automated using in‐house customized Pinnacle scripts. The automatic plans were generated for 20 patients using leave‐one‐out scheme and were evaluated against the corresponding clinical plans.

**Results:**

The automatic plans significantly reduced rectum and bladder $V_{50\;\;\mathrm{Gy}}$ by 11.79% ± 5.2% (*p* < 0.01) and 2.85% ± 3.16% (*p* < 0.01), respectively. The dose parameters achieved for the PTV and other OARs were comparable to those in the clinical plans. The entire planning process, including both dose prediction and inverse optimization, costs about 6 min.

**Conclusions:**

The direct 3D contour match method utilizes the full spatial information of the PTV and OARs of interest and provides an intuitive measurement for patient plan anatomy similarity. The proposed automatic planning method can generate plans with better quality and higher efficiency.

## 1 INTRODUCTION

Intensity‐modulated radiation therapy (IMRT) is a broadly utilized radiation treatment technique that can provide conformal coverage to the planning target volume (PTV) while sparing organs at risk (OARs). IMRT treatment planning usually starts with a universal template that specifies dose constraints for both the PTV and OARs following a given clinical protocol such as the often used Radiation Therapy Oncology Group protocols or institute‐specific guidelines. The planners often have to either tighten or loosen the dose constraints to adapt any individual patient's anatomy, particularly, the geometrical relationship between the PTV and OARs. To what extent the constraints are modified depends on the planner's experience. Therefore, the plan optimization step is a trial‐and‐error process in which the planner has to iteratively probe the achievable dose objectives until the plan satisfies the attending physician's specification. This process is tedious and time‐consuming, because it is difficult to tell when the plan quality is optimized and whether the optimization process should be stopped.

Various approaches, including knowledge‐based planning, atlas‐based planning, and deep learning methods, have been proposed to improve IMRT planning quality and efficiency.^1^, ^2^, ^3^, ^4^ In knowledge‐based planning approaches, the plan for a new patient is optimized using dose objectives achieved by previous clinical patients bearing similar PTV and OAR geometrical relationships. The geometric relationships are represented by one‐ (1D) or two‐dimensional (2D) metrics, including distance‐to‐target histogram,^5^, ^6^, ^7^ surface distance,^8^, ^9^ overlap volume histogram (OVH),^10^, ^11^, ^12^, ^13^, ^14^, ^15^, ^16^, ^17^ and contour beam's‐eye‐view (BEV) projection match.^18^, ^19^ However, the low‐dimensional metrics may oversimplify the three‐dimensional (3D) patient anatomy and result in a suboptimal selection of anatomically similar patient. Recently, deep learning methods also have been investigated to predict dose distribution^20^, ^21^, ^22^, ^23^, ^24^ and to automatically generate machine deliverable treatment plans,^25^, ^26^, ^27^, ^28^, ^29^ so as to improve the treatment planning quality and efficiency. The deep learning method, however, requires a sufficiently large dataset and a nontrivial network training process, which may have to be retrained once the dataset is expanded.

In this work, we proposed a knowledge‐based automatic treatment planning method using direct 3D patient anatomy match to select anatomically similar patients from an established plan database. The anatomical match was quantified with the degree of the PTV and OARs registration between a new patient and those in the plan database. The effectiveness of this method was validated on cervical cancer radiation therapy patients. This work is to provide an intuitive method to quantify the patient anatomical similarity and a practical approach for knowledge‐based automatic treatment planning.

## 2 METHODS

### 2.1 Cervical radiation therapy

We collected 81 clinical treatment plans for cervical cancer patients treated in the First Affiliated Hospital of USTC. A prescription dose of 50 Gy was delivered to the PTV in 25 fractions using seven 6‐MV photon beams with 180, 140, 90, 30, 330, 275, and 220° fixed beam angles. IMRT treatment plans were generated in a Pinnacle treatment planning system (Version 16.2, Philips Healthcare, Andover, MA). The PTV objectives were a uniform dose of 50 Gy, a minimum dose of 49.5 Gy, a maximum dose of 52 Gy, and at least 96% PTV volume receiving 50 Gy. The OARs considered for dose sparing were rectum, bladder, left and right femoral head, small intestine, and spinal cord. The dose objectives used in plan optimization are listed in Table 1. The dose goals used in plan evaluation are listed in Table 2. Specifically, the dose constraints for rectum and bladder were both $V_{50\;\;\mathrm{Gy}}<50\%$.

**TABLE 1:** Dose objectives in plan optimization

Structure	Parameter	Value
PTV	$\mathrm{Minimum}\;V_{50\;\;\mathrm{Gy}}$	96%
	$D_{max}$	52 Gy
	$D_{min}$	49.5 Gy
	Uniformdose	50 Gy
Rectum	$V_{45\;\;\mathrm{Gy}}$	45%
Bladder	$V_{45\;\;\mathrm{Gy}}$	45%
Left femoral head	$V_{45\;\;\mathrm{Gy}}$	5%
Right femoral head	$V_{45\;\;\mathrm{Gy}}$	5%
Small intestine	$V_{45\;\;\mathrm{Gy}}$	10%
	$D_{max}$	50 Gy
Spinal cord	$D_{max}$	42 Gy

Abbreviation: PTV, planning target volume.

**TABLE 2:** Dose goals in plan evaluation

**Structure**	**Parameter**	**Value**
PTV	$V_{50\;\;\mathrm{Gy}}$	>96%
Rectum	$V_{50\;\;\mathrm{Gy}}$	<50%
Bladder	$V_{50\;\mathrm{Gy}}$	<50%
Left femoral head	$V_{50\;\;\mathrm{Gy}}$	<10%
Right femoral head	$V_{50\;\;\mathrm{Gy}}$	<10%
Small intestine	$V_{50\;\;\mathrm{Gy}}$	<10%
	$D_{max}$	<52 Gy
Spinal cord	$D_{max}$	<45 Gy

Abbreviation: PTV, planning target volume.

Twenty patients were randomly selected from the database as test cases for automatic planning. Using the leave‐one‐out method, meaning each test case was compared against the remaining 80 patients in the database, the best anatomical match was identified. The automatic plans generated with the proposed method were compared with the corresponding clinical plans.

### 2.2 Direct 3D anatomy registration

**FIGURE 1:**
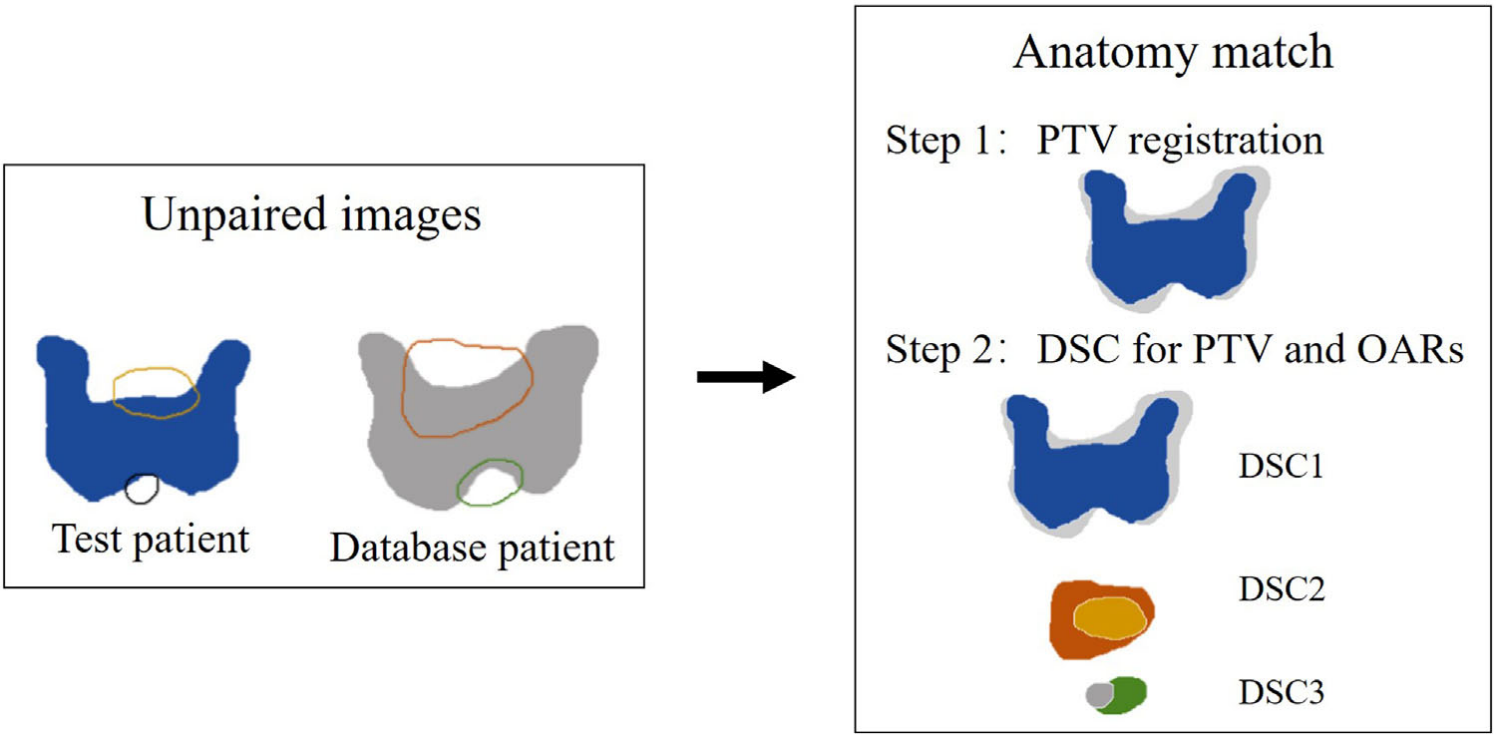
An anatomy match example of a test patient and a database patient. The PTV, rectum, and bladder of test and database case were used for patient anatomy match. There were two steps: step 1 was PTV registration, and step 2 was similarity measurement using DSC for the PTV, rectum, and bladder. DSC, Dice similarity coefficient; PTV, planning target volume

The best anatomical match between the test patient and the database patients was identified through rigid structure registration which, as shown in Figure 1, was a two‐step approach. First, the PTV was aligned to each case in the database by minimizing the pixel value difference using the squared error (SE) term:

(1) $$\begin{array}{c} \begin{array}{c} SE_{t,m}\left(u,v,w\right)=\sum_{x=0}^{M-1}\sum_{y=0}^{N-1}\sum_{z=0}^{L-1}\\ {\left\{I^{test}\left(x,y,z\right)-I^{match}\left(x+u,y+v,\;z+w\right)\right\}}^{2} \end{array} \end{array}$$

where $I^{test}$ and $I^{match}$ are the 3D binary intensities (1 inside PTV, 0 outside PTV) for the test and compared case, respectively.

The SE can be written as^30^, ^31^

(2) $$\begin{array}{c} SE_{t,m}\left(u,v,w\right)=C_{t}-2cor_{t,m}+C_{m} \end{array}$$

where

(3) $$\begin{array}{ccc} cor_{t,m} & = & \sum_{x=0}^{M-1}\sum_{y=0}^{N-1}\sum_{z=0}^{L-1}I^{test}\left(x,y,z\right)I^{match}\\ & & \left(x+u,y+v,z+w\right) \end{array}$$

(4) $$C_{t}=\sum_{x=0}^{M-1}\sum_{y=0}^{N-1}\sum_{z=0}^{L-1}{\left\{I^{test}\left(x,y,z\right)\right\}}^{2}$$

(5) $$\begin{array}{ccc} C_{m} & = & \sum_{x=0}^{M-1}\sum_{y=0}^{N-1}\sum_{z=0}^{L-1}{\left\{I^{match}\left(x+u,y+v,z+w\right)\right\}}^{2}\\ & & =\sum_{x=0}^{M-1}\sum_{y=0}^{N-1}\sum_{z=0}^{L-1}{\left\{I^{match}\left(x,y,z\right)\right\}}^{2} \end{array}$$

The $C_{t}$ and $C_{m}$ in Equation (2) are constants, independent of the candidate motion vector (u,v,w), and are not required to be computed, because we are interested only in finding u,v,andw where the SE function has minimum, not the actual minimum value itself. Thus, minimizing SE can be regarded as maximizing the following term:

(6) $$\begin{array}{ccc} S\left(u,v,w\right) & = & co\;r_{t,m}=\sum_{x=0}^{M-1}\sum_{y=0}^{N-1}\sum_{z=0}^{L-1}I^{test}\left(x,y,z\right)\\ & & \times \;I^{match}\left(x+u,y+v,z+w\right) \end{array}$$

The previous equation can be viewed as a spatial correlation between $I^{test}\;\mathrm{and}\;I^{match}$. Equation (3) can be also computed using the fast Fourier transform (FFT) algorithm as follows:

(7) $$\begin{array}{c} \begin{array}{c} S\left(u,v,w\right)=\mathrm{IFFT}\left[\mathrm{FFT}\times \left(I^{\mathrm{test}}\right)\mathrm{FFT}\left(I^{\mathrm{match}}\right)\right] \end{array} \end{array}$$

where IFFT is the inverse FFT, and the asterisk indicates the complex conjugation. To perform multiplication in the transform domain, the images must be padded with zeros up to the same size. To improve computing efficiency, parallel computing was used on a MATLAB platform.

Second, the similarities of PTV, rectum, and bladder between test and each compared patient were evaluated by Dice similarity coefficient (DSC) after the PTV was registered. The DSC equation was

(8) $$\begin{array}{c} DSC=2\times \frac{I^{test}\cap I^{match}}{I^{test}+I^{match}} \end{array}$$

Then, the best anatomical match was selected based on the DSC value. For the PTV similarity match, 20 patients with highest DSC scores were chosen for subsequent OAR similarity match. After that, three patients with highest DSC scores for each OAR were selected, and the smallest dose–volume histogram (DVH) parameter $V_{50\;\;\mathrm{Gy}}$ of the three was used as the achievable dose constraint in the automatic planning process. The dose optimization objectives for the rest of OARs were from our institute protocol as shown in Table 1.

### 2.3 Automatic treatment planning

For an incoming patient, the automatic planning process follows five steps: (1) The patient PTV, rectum, and bladder contours were extracted; (2) the patient was registered with the database based on the PTV structure; (3) the best anatomical match patients were found and the dose constraints were derived; (4) an initial plan was automatically generated and the personalized objectives were applied; and (5) start plan optimization.

**FIGURE 2:**
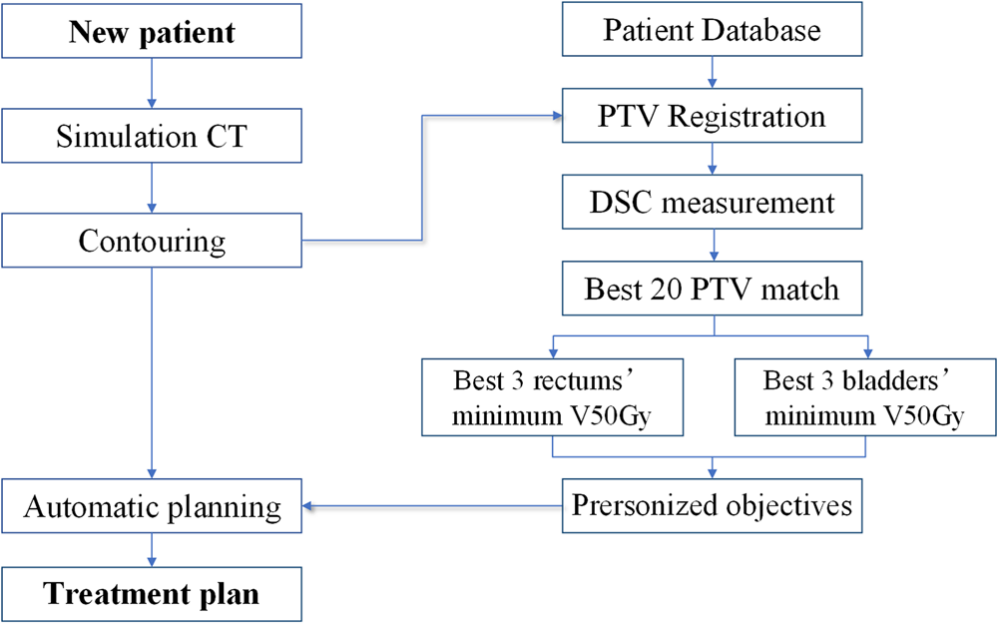
Automatic planning flowchart

We developed a series of scripts to further automate the execution procedures. The scripts are applied to (1) generate the ring structures surrounding the PTV and spinal cord to limit the dose falloff outside the PTV and OAR, (2) set seven radiation beams, and (3) apply all PTV and OAR dose objectives. An example workflow of automatic planning is shown in Figure 2.

### 2.4 Plan data analysis

The automatic plan was compared with its clinical counterpart for the evaluation of achieved target coverage and OARs sparing. The homogeneity indices (*HI*), $D_{98},\;D_{95},\;D_{2},\;\mathrm{and}\;V_{50\;\;\mathrm{Gy}}$, were used for target coverage evaluation. The definition of *HI* is as follows:

(9) $$\begin{array}{c} HI=\frac{D_{2}-D_{98}}{D_{Rx}}\times 100\% \end{array}$$

where $D_{2}\;\mathrm{and}\;D_{98}$ are the dose value corresponding to 2% and 98% of the PTV on the DVH, respectively, and $D_{Rx}$ is the prescription dose 50 Gy. The dose parameters used in comparison included: $V_{50\;\;\mathrm{Gy}}$ for rectum and bladder, $V_{50\;\;\mathrm{Gy}}$ for left and right femoral head, $V_{50\;\;\mathrm{Gy}}$ and max dose for small intestine, max dose for spinal cord.

The paired *t*‐test was used for comparison with significance determined at the level of *p* < 0.05.

## 3 RESULTS

**FIGURE 3:**
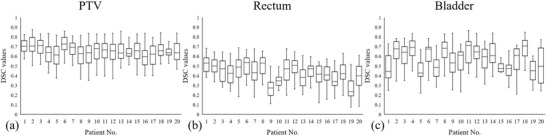
Box plot of DSC distributions in similarity match for the 20 test patients: (a) The PTV DSC distribution against the remaining 80 patients in the database. (b) The rectum DSC distribution against the top 20 PTV‐similar patients. (c) The bladder DSC distribution against the top 20 PTV‐similar patients. DSC, Dice similarity coefficient; PTV, planning target volume

The PTV DSC distributions against remaining 80 patients in the database for 20 test patients are shown in Figure 3a. The average median DSC was 0.66 ± 0.04, with a highest median DSC of 0.73 and a lowest median DSC of 0.60. Considering the limited database size, we empirically chose the top 20 patients in the PTV match instead of setting a constant Dice threshold. The rectum and bladder DSC distributions for the chosen 20 patients are shown in Figure 3b,c. The average median DSC for rectum and bladder were 0.43 ± 0.08 and 0.58 ± 0.10, respectively. The top three patients with highest rectum or bladder DSC values were selected as best matched patients for the following dose constraint determination. Each test patient's DSC results used for anatomically similar patient selection are listed in Table 3. The results indicate that the rigid structure registration is capable of identifying anatomically similar patients from which the DVH parameters were extracted for automatic planning.

**TABLE 3:** The DSC results in anatomically similar patient selection

**Pt. No**.	**20 PTV DSC**	**3 Rectum DSC**	**3 Bladder DSC**	**6 PTV DSC**
**1**	0.79 ± 0.02	0.65 ± 0.02	0.69 ± 0.03	0.79 ± 0.01
**2**	0.82 ± 0.02	0.61 ± 0.03	0.78 ± 0.01	0.83 ± 0.03
**3**	0.79 ± 0.02	0.63 ± 0.01	0.77 ± 0.03	0.78 ± 0.01
**4**	0.73 ± 0.03	0.56 ± 0.05	0.80 ± 0.03	0.73 ± 0.03
**5**	0.75 ± 0.03	0.65 ± 0.04	0.59 ± 0.07	0.75 ± 0.02
**6**	0.82 ± 0.02	0.65 ± 0.02	0.74 ± 0.04	0.83 ± 0.02
**7**	0.77 ± 0.02	0.61 ± 0.06	0.66 ± 0.06	0.78 ± 0.02
**8**	0.74 ± 0.03	0.64 ± 0.01	0.81 ± 0.02	0.74 ± 0.01
**9**	0.75 ± 0.04	0.41 ± 0.07	0.75 ± 0.08	0.80 ± 0.03
**10**	0.77 ± 0.03	0.48 ± 0.04	0.71 ± 0.01	0.80 ± 0.02
**11**	0.78 ± 0.03	0.62 ± 0.05	0.81 ± 0.05	0.80 ± 0.01
**12**	0.78 ± 0.04	0.60 ± 0.04	0.78 ± 0.05	0.78 ± 0.03
**13**	0.79 ± 0.03	0.54 ± 0.06	0.75 ± 0.00	0.80 ± 0.04
**14**	0.74 ± 0.04	0.58 ± 0.02	0.78 ± 0.05	0.76 ± 0.05
**15**	0.78 ± 0.03	0.53 ± 0.01	0.62 ± 0.08	0.78 ± 0.03
**16**	0.73 ± 0.05	0.54 ± 0.02	0.60 ± 0.05	0.74 ± 0.03
**17**	0.75 ± 0.03	0.51 ± 0.04	0.71 ± 0.01	0.76 ± 0.02
**18**	0.78 ± 0.03	0.57 ± 0.06	0.80 ± 0.04	0.78 ± 0.03
**19**	0.71 ± 0.04	0.48 ± 0.06	0.59 ± 0.01	0.75 ± 0.03
**20**	0.79 ± 0.03	0.59 ± 0.02	0.74 ± 0.04	0.79 ± 0.03
Mean ± SD	**0.76** ± **0.04**	**0.57** ± **0.07**	**0.72** ± **0.08**	**0.78** ± **0.38**
Median	**0.77**	**0.59**	**0.75**	**0.78**

Abbreviations: DSC, Dice similarity coefficient; PTV, planning target volume.

**FIGURE 4:**
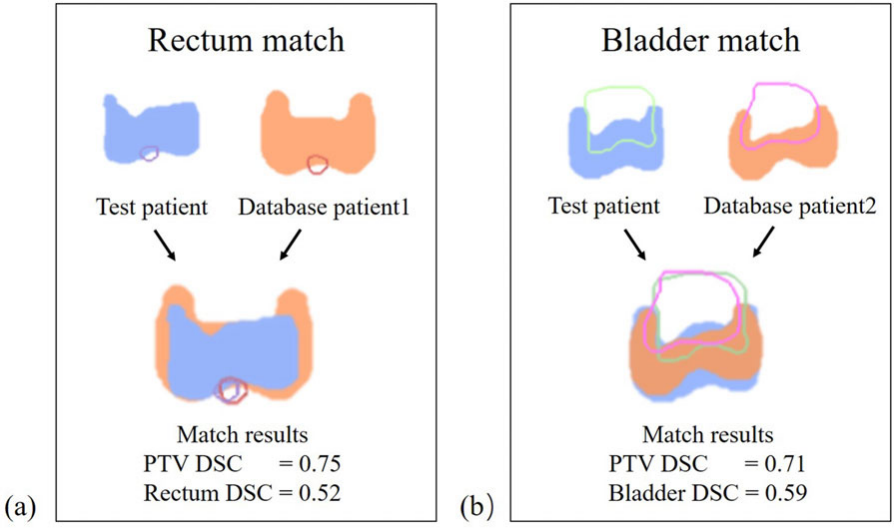
An example of the anatomical similarity for rectum and bladder: (a) The most similar patient for rectum dose prediction; (b) the most similar patient for bladder dose prediction

**FIGURE 5:**
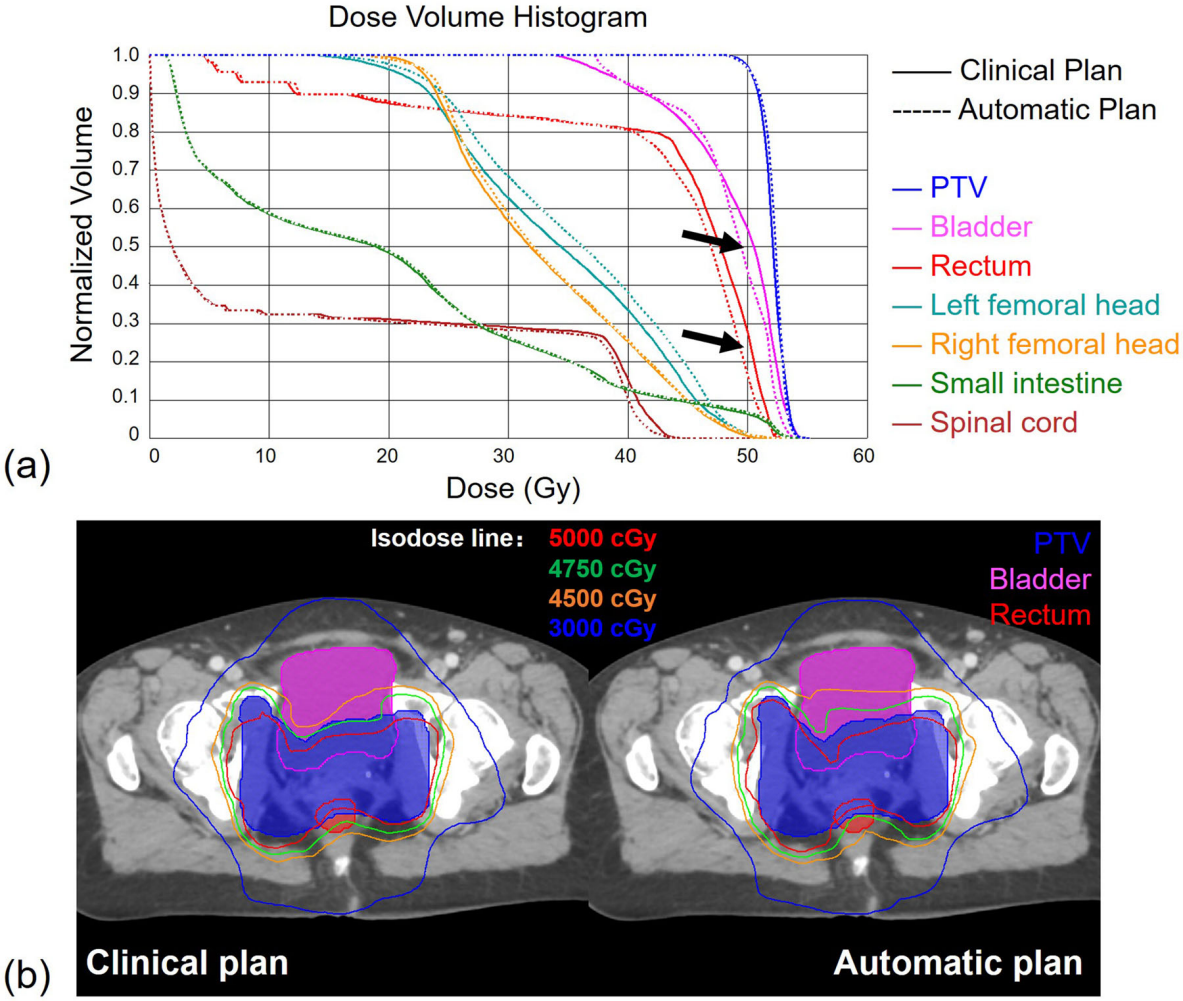
An example for the clinical and automatic treatment plan comparison: (a) DVHs of PTV, rectum, bladder, femoral heads, small intestine, and spinal cord; (b) dose distributions of the two plans. DVHs, dose–volume histograms; PTV, planning target volume

As an example, Figure 4 illustrates the anatomy registration results of one patient. The best matched patients’ $V_{50\;\;\mathrm{Gy}}$ dose constraints were used as automatic plan optimization objectives. Figure 5 compares the DVHs and dose distributions for the automatic and clinical plan of this patient. The PTV (dark blue) $V_{50\;\;\mathrm{Gy}}$ coverage of both plans meets the protocol (clinical 96.95% vs. automatic 96.54%). The DVH curves for the PTV, right femoral head, and small intestine are very similar between the two plans. The DVHs for the rectum, bladder, and spinal cord are slightly better at the high‐dose region, whereas the DVH for the left femoral head is slightly worse in the automatic plan.

Table 4 compares the PTV dose metrics between the clinical and automatic plans. The automatic plans achieved an average PTV $V_{50\;\;\mathrm{Gy}}$ of 97.3% ± 0.62%, whereas the clinical plans achieved an average of 97.03% ± 0.55%, with no significant difference in between (*p* = 0.14). The automatic plans were significantly lower than clinical plans in $D_{98}$ (*p* = 0.03), although the average dose difference was only 0.15 ± 0.29 Gy. Moreover, there is no significant difference for $D_{95},\;D_{2},\;\mathrm{and}\;HI$ values between the two groups. These imply that both sets of plans achieved similar PTV coverage.

**TABLE 4:** Comparison of the PTV/OAR dose metrics between the clinical and automatic plans

**Structures**	**Metrics**	**Automatic plan**	**Clinical plan**	***p*‐Value**
PTV	$V_{50}\left(\%\right)$	97.03 ± 0.55	97.30 ± 0.62	0.14
	HI(%)	8.00 ± 1.00	8.00 ± 1.00	0.25
	$D_{98}\left(Gy\right)$	49.63 ± 0.21	49.78 ± 0.19	0.03
	$D_{95}\left(Gy\right)$	50.50 ± 0.15	50.51 ± 0.19	0.82
	$D_{2}\left(Gy\right)$	53.73 ± 0.21	53.78 ± 0.25	0.31
Rectum	$V_{50}\left(\%\right)$	23.22 ± 6.20	35.01 ± 5.67	**<0.001**
Bladder	$V_{50}\left(\%\right)$	36.43 ± 4.62	39.28 ± 5.61	**0.001**
Left femoral head	$V_{50}\left(\%\right)$	1.98 ± 2.00	1.56 ± 1.08	0.31
Right femoral head	$V_{50}\left(\%\right)$	1.42 ± 0.93	1.73 ± 1.00	**0.02**
Small intestine	$V_{50}\left(\%\right)$	8.69 ± 3.64	8.30 ± 3.72	**0.02**
	$D_{max}\left(Gy\right)$	53.86 ± 0.41	53.84 ± 0.37	0.90
Spinal cord	$D_{max}\left(Gy\right)$	43.99 ± 0.60	43.65 ± 0.79	**0.02**

*Note*: Table values bolded when statistically significant (*p* < 0.05).

Abbreviations: *HI*, homogeneity index; OAR, organs at risks; PTV, planning target volume.

**FIGURE 6:**
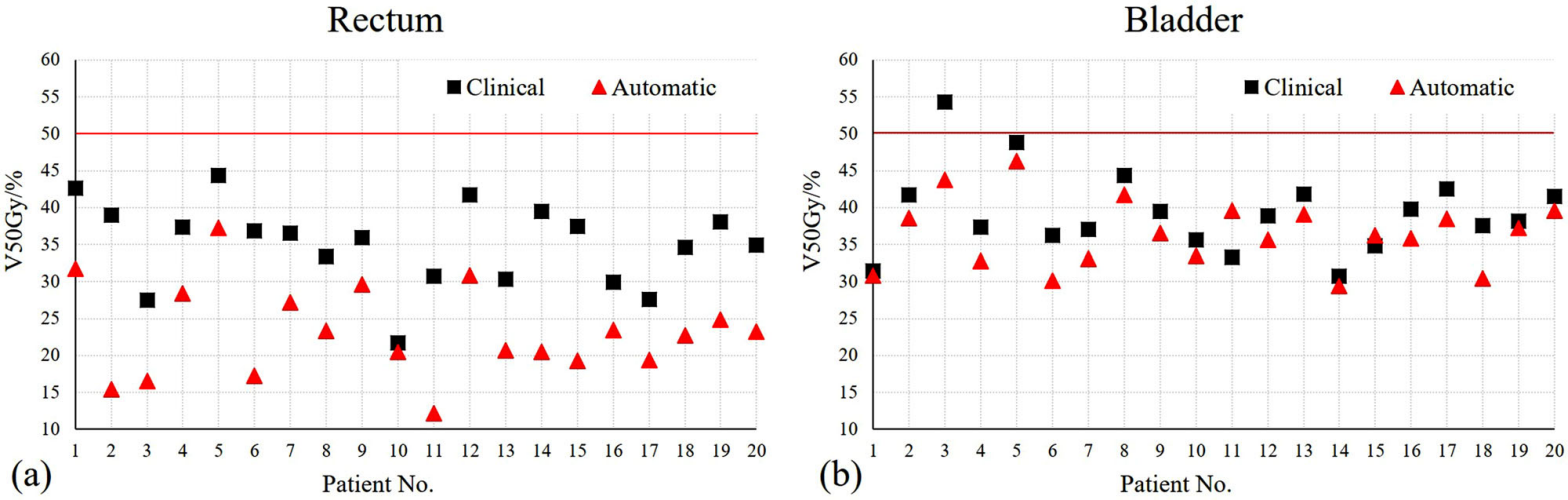
Automate planning results of 20 patients compared with clinical ones. The red triangle markers are the automatic plan results, and the black square markers are the clinical results: (a) the rectum $V_{50\;\;\mathrm{Gy}}$ of 20 patients; (b) the bladder $V_{50\;\;\mathrm{Gy}}$ of 20 patients

The dose comparisons for OARs are provided in Table 4. The rectum $V_{50\;\;\mathrm{Gy}}$ in the automatic plans was significantly reduced by 11.79% ± 5.46% (23.22% ± 6.2% vs. 35.01% ± 5.67%, *p* < 0.001). The bladder $V_{50\;\;\mathrm{Gy}}$ in the automatic plans was significantly reduced by 2.85% ± 3.33% (36.43% ± 4.62% vs. 39.28% ± 5.61%, *p* = 0.001). For the rest OARs, the value differences for the evaluated dose parameters were relatively small and were within 0.5% or 0.5 Gy. As observed in Figure 6, the $V_{50\;\;\mathrm{Gy}}$ values for the rectum and bladder were reduced in the automatic plans for almost all 20 test patients (100% for rectum, and 90% for bladder). The spinal cords $D_{max}$ for automatic plans were significantly higher than the clinical plans. However, all the max doses for spinal cord were under 45 Gy.

For each new patient, the registration process took an average of 2 min. In addition, the treatment plan optimization took an average of 4 min. For each test patient, the entire automatic planning process took about 6 min.

## 4 DISCUSSION

We have developed a direct 3D anatomy match method to search geometrically similar patients from a database base on the proposed structure similarity. The achievable dose constraints for a new patient were obtained from those clinical plans in the database corresponding to anatomically most similar patients and applied as personalized optimization objectives. In addition, we developed a workflow to automate the planning process and minimized the number of manual interactions. The validation results show that the plans created with the proposed method had quality comparable to their clinical counterparts.

The direct 3D anatomy match measures the similarity between patients using their original volume and location, which preserves both the geometric relationship and anatomical features of the PTV and OARs as much as possible. In the 1D OVH method,^10^, ^12^ the absolute volume of either PTV or OARs is not considered. In addition, the relative direction between the PTV and OAR is not considered either. In the 2D BEV projection method,^18^, ^19^ the depth information, that is, the closeness between the PTV and OAR along the beam direction, is not considered.

Data‐searching efficiency is critical in database‐associated knowledge‐based planning. There is a tradeoff between the searching efficiency and the completeness of data used in patient anatomy match. In this study, a feature‐based rigid registration method is developed where the PTV and key OARs are used as the alignment landmark. This method to a large extent reduces the unessential anatomy information for patient match otherwise employed in image‐based registration and, thus, improves the searching efficiency. Besides, as we want to quantify the actual differences in geometrical relationships between the two patients, including the rotational difference, only a translation registration was used and reduces computational difficulty. Furthermore, the feature registration is optimized with an FFT‐based technique that dramatically enhances the calculation speed. Using the current program developed in MATLAB, the patient searching through the database of 80 patients takes about 2 min. In future, parallel computing with GPU can further accelerate the patient searching process to the order of 1 s or even less.

This study has several limitations. First, like all other KBP methods, the quality of the automatic plan is determined by both the diversity of the patient anatomy and the quality of the clinical plans in the database. Currently, the number of patients included in the database is 81 and is relatively small, which may render out suboptimal patient anatomy match. So, we chose to select a similar rectum and bladder independently instead of in combination for more strict constraints to improve the plan quality as much as possible. In the future, we would expand our patient database and examine the results difference when combined evaluation is used in similarity search. Despite the small number, the database can be improved through self‐iteration by updating each individual plan against the rest plans with the leave‐one‐out strategy. To keep improving the database, it may be decided that only the new plan with quality better than its anatomically matched counterpart in the database can be included. Regardless, how to quantify the diversity of patient anatomy in a database is still an open question. Moreover, the method is tested on 7‐field IMRT plans on cervical cancer patients that are relatively simple. VMAT plans and other treatment sites should also be tested in the future. In addition, it is worth noting that this method is not intended to generate a plan with best quality. Rather, it aims to achieve the best quality that has been achieved by the anatomically similar patients in the database.

## 5 CONCLUSIONS

An automatic treatment planning method based on direct 3D patient anatomy match has been developed and validated on cervical cancer IMRT. Using an FFT‐accelerated PTV registration method, the anatomically similar patients could be selected from a database containing 80 patients in around 2 min. Taking the dose parameters achieved in the anatomically similar patients as the dose constraints for the new patient, this automatic planning method improves the planning efficiency without compromising the plan quality. The rectum and bladder doses were significantly reduced for the automatic plans compared to their clinical counterparts.

## CONFLICT OF INTEREST

The authors declare no conflict of interest.

## AUTHOR CONTRIBUTIONS

Duoer Zhang: Study design, data analysis and manuscript drafting; Zengtai Yuan: Data collection and manuscript revision; Panpan Hu: Data collection and manuscript revision; Yidong Yang: Study guidance, manuscript revision and financial support.
